# Heterogeneity of intratumoral ^111^In-ibritumomab tiuxetan and ^18^F-FDG distribution in association with therapeutic response in radioimmunotherapy for B-cell non-Hodgkin’s lymphoma

**DOI:** 10.1186/s13550-015-0093-3

**Published:** 2015-03-14

**Authors:** Kohei Hanaoka, Makoto Hosono, Yoichi Tatsumi, Kazunari Ishii, Sung-Woon Im, Norio Tsuchiya, Kenta Sakaguchi, Itaru Matsumura

**Affiliations:** Department of Radiology, Faculty of Medicine, Kinki University, 377-2 Ohno-Higashi, Osaka-Sayama, 589-8511 Japan; Division of Positron Emission Tomography, Institute of Advanced Clinical Medicine, Faculty of Medicine, Kinki University, 377-2 Ohno-Higashi, Osaka-Sayama, 589-8511 Japan; Department of Hematology, Faculty of Medicine, Kinki University, 377-2 Ohno-Higashi, Osaka-Sayama, 589-8511 Japan; Neurocognitive Disorders Center, Faculty of Medicine, Kinki University, 377-2 Ohno-Higashi, Osaka-Sayama, 589-8511 Japan

**Keywords:** Radioimmunotherapy, Heterogeneity, Ibritumomab tiuxetan, FDG, PET/CT, SPECT/CT

## Abstract

**Background:**

The purpose of this study was to quantitatively evaluate the tumor accumulation and heterogeneity of ^111^In-ibritumomab tiuxetan (Zevalin®) and tumor accumulation of ^18^F-fluoro-deoxyglucose (FDG) and compare them to the tumor response in B-cell non-Hodgkin’s lymphoma patients receiving ^90^Y-ibritumomab tiuxetan (Zevalin®) therapy.

**Methods:**

Sixteen patients with histologically confirmed non-Hodgkin’s B-cell lymphoma who underwent ^90^Y-ibritumomab tiuxetan therapy along with ^111^In-ibritumomab tiuxetan single-photon emission computerized tomography (SPECT)/CT and FDG positron emission tomography (PET)/CT were enrolled in this retrospective study. On pretherapeutic FDG PET/CT images, the maximum standardized uptake value (SUVmax) was measured. On SPECT/CT images, a percentage of the injected dose per gram (%ID/g) and SUVmax of ^111^In-ibritumomab tiuxetan were measured at 48 h after its administration. The skewness and kurtosis of the voxel distribution were calculated to evaluate the intratumoral heterogeneity of tumor accumulation. As another intratumoral heterogeneity index, cumulative SUV-volume histograms describing the percentage of the total tumor volume above the percentage thresholds of pretherapeutic FDG and ^111^In-ibritumomab tiuxetan SUVmax (area under the curve of the cumulative SUV histograms (AUC-CSH)) were calculated. All lesions (*n* = 42) were classified into responders and non-responders lesion-by-lesion on pre- and post-therapeutic CT images.

**Results:**

A positive correlation was observed between the FDG SUVmax and accumulation of ^111^In-ibritumomab tiuxetan in lesions. A significant difference in pretherapeutic FDG SUVmax was observed between responders and non-responders, while no significant difference in ^111^In-ibritumomab tiuxetan SUVmax was observed between the two groups. In contrast, voxel distribution of FDG demonstrated no significant differences in the three heterogeneity indices between responders and non-responders, while ^111^In-ibritumomab tiuxetan demonstrated skewness of 0.58 ± 0.16 and 0.73 ± 0.24 (*p* < 0.05), kurtosis of 2.39 ± 0.32 and 2.78 ± 0.53 (*p* < 0.02), and AUC-CSH of 0.37 ± 0.04 and 0.34 ± 0.05 (*p* < 0.05) for responders and non-responders.

**Conclusions:**

Pretherapeutic FDG accumulation was predictive of the tumor response in ^90^Y-ibritumomab tiuxetan therapy. The heterogeneity of the intratumoral distribution rather than the absolute level of ^111^In-ibritumomab tiuxetan was correlated with the tumor response.

## Background

Ibritumomab tiuxetan is a CD20-directed radiotherapeutic antibody administered as part of the therapeutic regimen indicated for the treatment of patients with relapsed or refractory, low-grade, or follicular B-cell non-Hodgkin’s lymphoma [1,2].

Prior to ^90^Y-ibritumomab tiuxetan (^90^Y-Zevalin®) therapy, imaging with ^111^In-ibritumomab tiuxetan (^111^In-Zevalin®) is performed according to a therapy protocol implemented in certain countries and regions to verify the expected biodistribution and exclude patients who show an altered biodistribution, such as the rapid clearance of ^111^In-Ibritumomab tiuxetan from the blood pool, with prominent liver, spleen, or marrow uptake [3,4]. Such criteria for expected and altered biodistributions have been proposed and established, based on which the indication of radioimmunotherapy with ^90^Y-ibritumomab tiuxetan is assessed. A high rate of a complete response after ^90^Y-ibritumomab tiuxetan therapy has often been observed in patients with negative ^111^In-ibritumomab tiuxetan accumulation in lesions [5]. It has been speculated that non-uniformity in the intratumorally absorbed dose plays a significant role in the success or failure of radionuclide therapy [6-9]. Thus, the association between the tumor response and ^111^In-ibritumomab tiuxetan accumulation in lesions should be clarified. For this purpose, single-photon emission computerized tomography (SPECT)/CT may have advantages over whole-body planar scans because it provides three-dimensional images by fusing data on function and morphology.

^18^F-fluoro-deoxyglucose (FDG) positron emission tomography (PET) is another imaging modality often used during the course of ^90^Y-ibritumomab tiuxetan therapy [10,11]. This noninvasive, three-dimensional imaging modality has become widely used and essential for the initial staging and evaluation of the response after treatment in patients with malignant lymphoma and has been integrated in the Revised International Workshop criteria for malignant lymphoma [12]. The role of FDG-PET for predicting outcomes after ^90^Y-ibritumomab tiuxetan therapy has been reported [13,14], and a lower pretherapeutic FDG uptake may correlate with a longer progression-free survival [11].

In this study, using SPECT/CT, we measured absolute levels of ^111^In-ibritumomab tiuxetan accumulation and assessed the heterogeneity of ^111^In-ibritumomab tiuxetan distribution in lesions by calculating heterogeneity indices. We focused on follicular lymphoma and clarified the tumor accumulation and intratumoral heterogeneity of ^111^In-ibritumomab tiuxetan on SPECT/CT and tumor accumulation of FDG on PET/CT lesion-by-lesion and compared them to the tumor response after ^90^Y-ibritumomab tiuxetan therapy.

## Methods

### Patients

Thirty-seven patients with histologically confirmed follicular lymphoma underwent ^90^Y-ibritumomab tiuxetan therapy during the period between January 2009 and December 2012 in our hospital. Of these 37 patients, 16 met the following criteria: they underwent (a) pretherapeutic PET/CT, (b) post-therapeutic CT, and (c) pretherapeutic ^111^In-ibritumomab tiuxetan SPECT/CT along with whole-body planar scans and (d) they had at least one lymphoma lesion analyzable on images as a target lesion, that is, 1 cm or more in diameter. The 16 patients (6 females and 10 males, mean age: 67.3 ± 7.5 years, range: 49 to 79 years) enrolled in this study are presented in Table 1. The numbers of patients with Ann Arbor stages 1, 2, 3, and 4 were 4, 2, 5, and 5, respectively. The PET/CT and SPECT/CT examinations of these patients were analyzed based on the tumor response. The institutional review board waived the requirement of the informed consent of patients and approved this retrospective study.

**Table 1:** **Characteristics of all 16 patients investigated in this study**

**Patient**	**Age**	**Gender**	**Body mass (kg)**	**Initial stage**	**Number of regimen**	**Time between pre-therapeutic PET and Zevalin therapy (days)**	**Time between Zevalin therapy and post-therapeutic CT (days)**	**Number of lesions** ^**a**^	**Response classification** ^**b**^
1	49	M	93.4	IIIb	1	23	65	2	CRu
2	74	M	63.4	IIIa	2	4	63	2	CR
3	67	F	50.6	II	4	57	91	4	PR
4	61	F	54.6	I	1	50	78	1	CR
5	58	M	73.6	I	1	55	58	1	CR
6	72	M	66.4	IV	2	71	68	7	CR
7	71	F	57.7	IV	2	58	75	5	CR
8	73	M	63.4	II	3	14	64	4	PD
9	66	M	62.9	IIIa	3	33	62	6	SD
10	71	M	53.6	I	2	42	77	1	SD
11	68	F	49.1	IV	3	27	62	1	CR
12	79	F	43.0	IV	1	53	65	1	SD
13	61	F	60.7	I	2	41	92	1	PD
14	76	M	57.3	III	1	50	99	1	CR
15	65	M	50.7	IV	1	46	62	4	CR
16	66	M	49.0	III	1	15	75	1	PR

^a^Lesions analyzed in this study; ^b^based on the International Workshop Criteria (IWC). CR, complete response; CRu, unconfirmed complete response; PD, progressive disease; PR, partial response.

The protocol follows the ibritumomab tiuxetan therapeutic regimen described in the ibritumomab tiuxetan-prescribing information. Rituximab at 250 mg/m^2^ was infused over 4 h, followed by a 10-min infusion of 14.8 MBq/kg of ^90^Y-ibritumomab tiuxetan, not exceeding 1,184 MBq. One week before therapy, each patient received a similar infusion of rituximab followed by 185 MBq of ^111^In-ibritumomab tiuxetan. Patients received ^90^Y-ibritumomab tiuxetan therapy on the condition that they had the expected biodistribution on whole-body planar ^111^In-ibritumomab tiuxetan scans.

### PET/CT procedure

After fasting for at least 4 h, each patient was infused with 90 to 210 MBq of FDG (adjusted for body weight) intravenously for more than 2 min. After uptake for 60 min, PET/CT imaging was performed on a Biograph Duo (Siemens AG, Erlangen, Germany), which had a transaxial in-plane resolution of 6.2 mm. Whole-body PET was acquired for 110 s per bed position, and the number of positions was based on the patient’s height. The patients were asked to breathe normally during PET acquisition. PET data were collected in three-dimensional imaging mode and reconstructed using a CT transmission map for attenuation correction with the ordered subsets expectation maximization (OSEM) algorithm (two iterations, eight subsets) and a 5-mm Gaussian filter.

### Planar and SPECT/CT procedure

A SPECT scan was carried out for 12 min (continuous mode, 30 steps, magnification × 1.00, 128 × 128 matrix) in a resting, supine position, 48 h after the infusion of 185 MBq of ^111^In-ibritumomab tiuxetan. All SPECT/CT studies were performed using a rotating dual-headed gamma camera (Symbia T6, Siemens AG, Erlangen, Germany) with low-medium energy, general purpose (LMEGP) collimators. Energy windows were 172 keV: 15% width for the lower photopeak with lower and upper scatter windows of 15% and 8%, respectively, and 247 keV: 15% width for the upper photopeak with a lower scatter window of 10%. The CT scan was acquired in a helical mode with a voltage of 130 kV. CT slices were reconstructed at 5 mm (for SPECT attenuation correction) and 3 mm (for estimating the volume of the tumors).

All SPECT/CT data were reconstructed using the OSEM method with depth-dependent three-dimensional (transversal and axial) resolution recovery (OSEM-3D) (Flash3D; Siemens AG, Erlangen, Germany) and CT-based attenuation correction and energy window-based scatter correction. Subsets and iterations were six and nine, respectively. A Gaussian filter of 9 mm was used in order to reduce sensitivity to noise. Attenuation correction was performed using CT. Reconstructed images had a pixel size of 4.8 mm and slice thickness of 5 mm.

Whole-body anterior/posterior planar images were acquired at 1, 24, 48, and 72 h post-administration using a gamma camera (Symbia T6, Siemens AG, Erlangen, Germany) with LMEGP collimators. Gamma camera settings were: 256 × 1,024 matrix; dual energy photopeaks set at 172 and 247 keV; 15% symmetric window; scan speed of 10 cm/min. All 16 patients analyzed in this study had the expected biodistribution on these planar images according to the criteria [4] because it was a requirement for the subsequent radioimmunotherapy with ^90^Y-ibritumomab tiuxetan.

### Cross-calibration of SPECT/CT

Calibration was the process of establishing the relationship between the measured count rate per volume and true activity concentration. SPECT/CT images of the phantom with uniform activity in the entire intracranial cavity were used to calculate a cross-calibration factor for the relative sensitivity of the SPECT/CT scanner and well counter. The cross-calibration factor values were determined for reconstruction with the OSEM-3D method.

### Data analysis

An e.soft workstation (Siemens AG, Erlangen, Germany) was used as an analysis tool. On FDG PET/CT images, maximum standardized uptake value (SUVmax) of FDG was measured by placing a volumetric region of interest (VOI) over the tumor on PET images by semiautomatic contouring method using threshold of SUVmax referring to CT images [15]. On SPECT/CT images, tumor accumulations expressed as SUVmax and a percentage of the injected dose per gram (%ID/g) of ^111^In-ibritumomab tiuxetan was measured by referring to CT images.

In the 16 patients enrolled in this study, 42 lymphoma lesions of 1 cm or more were selected as analyzable. This cutoff value of 1 cm was determined in consideration of the spatial resolution of SPECT [16] and PET [17]. All lesions were classified into responders and non-responders by applying the ‘International Workshop Criteria’ [18] to each lesion on pre- and post-therapeutic CT images. Here, ‘International Workshop Criteria’ comprises a patient-by-patient therapeutic response standard, but we applied the criteria to evaluate the lesion-by-lesion therapeutic response. Another statistical approach taken to characterize lesion uptake was quantification of the spatial heterogeneity of voxel-based activities in histograms. The common quantifiers of skewness and kurtosis, describing the asymmetry and extent of symmetrical departure, respectively, were employed [19,20]. The skewness and the kurtosis of pretherapeutic FDG and ^111^In-ibritumomab tiuxetan were calculated according to the following formula:

$$ \mathrm{Skewness}=\frac{1}{n}{\displaystyle \sum_{i=1}^n{\left({x}_i-\overline{x}\right)}^3/}{s}^3 $$

$$ \mathrm{Kurtosis}=\frac{1}{n}{\displaystyle \sum_{i=1}^n{\left({x}_i-\overline{x}\right)}^4/}{s}^4 $$

where *n* is the number of voxels in the volume of interest, $\overline{x}$ is the mean value of total counts, and *s* is the standard deviation of total counts. In addition, cumulative SUV histograms (CSH) were used in order to characterize the heterogeneity of the intratumoral uptake of FDG and ^111^In-ibritumomab tiuxetan. In CSH, the percent volume of a tumor was plotted against a threshold value varying from 0 to 100% of SUVmax of the tumor, and the area under the curve of the cumulative SUV histograms (AUC-CSH) was assessed as a heterogeneity index. A lower AUC-CSH was assumed to correspond to a more heterogeneous distribution [21].

### Statistics

All parameters of the responder and non-responder lesions were compared with the Mann-Whitney *U* test. The Pearson product-moment correction coefficient was calculated between glucose metabolism and ^111^In-ibritumomab tiuxetan accumulation. All statistical analyses were performed with SPSS software (Version 17, SPSS Inc., Chicago, Illinois, USA), and a *p* value of less than 0.05 indicated significant differences.

## Results

**Figure 1:**
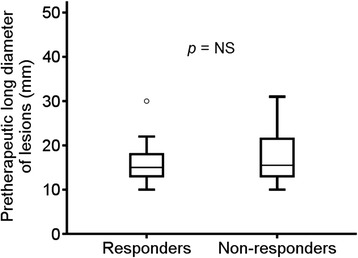
**Comparison of the diameter (mm) of pretherapeutic lesions between responders and non-responders.** The median value and the interquartile range are represented by box plot.

The 42 lesions of the 16 follicular lymphoma patients were classified into responder lesions (responders, complete response: 22, unconfirmed complete response: 2, partial response: 2) and non-responder lesions (non-responders, stable disease: 14, progressive disease: 2) on a lesion-by-lesion basis by referring to CT findings. The long diameter of pretherapeutic lesions was 15.9 ± 4.2 mm and 17.9 ± 6.4 mm for responders and non-responders, respectively. There was a tendency for responders to show a smaller diameter than non-responders, but no significant difference was observed (Figure 1).

**Figure 2:**
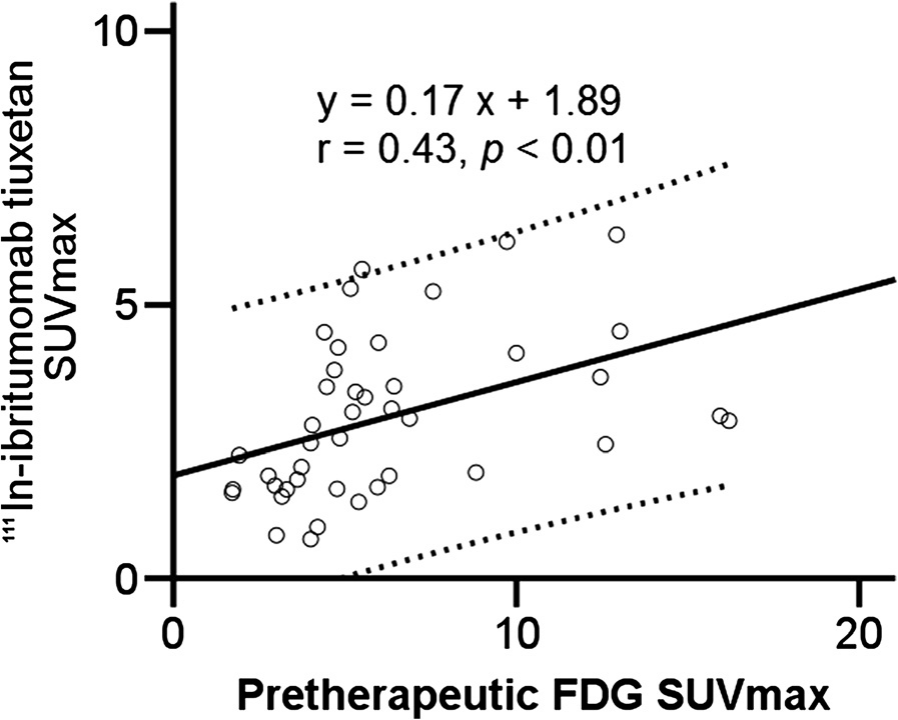
**Correlation between pretherapeutic FDG SUVmax and** ^**111**^ **In-ibritumomab tiuxetan SUVmax in all lesions.** The solid line is the progression line. Dotted lines represent 95% confidence intervals of the progression line.

There was a positive correlation between glucose metabolism and ^111^In-ibritumomab tiuxetan accumulation in lesions, showing regression lines of *y* = 0.17*x* + 1.89 (*r* = 0.43, *p* < 0.01) (Figure 2).

**Figure 3:**
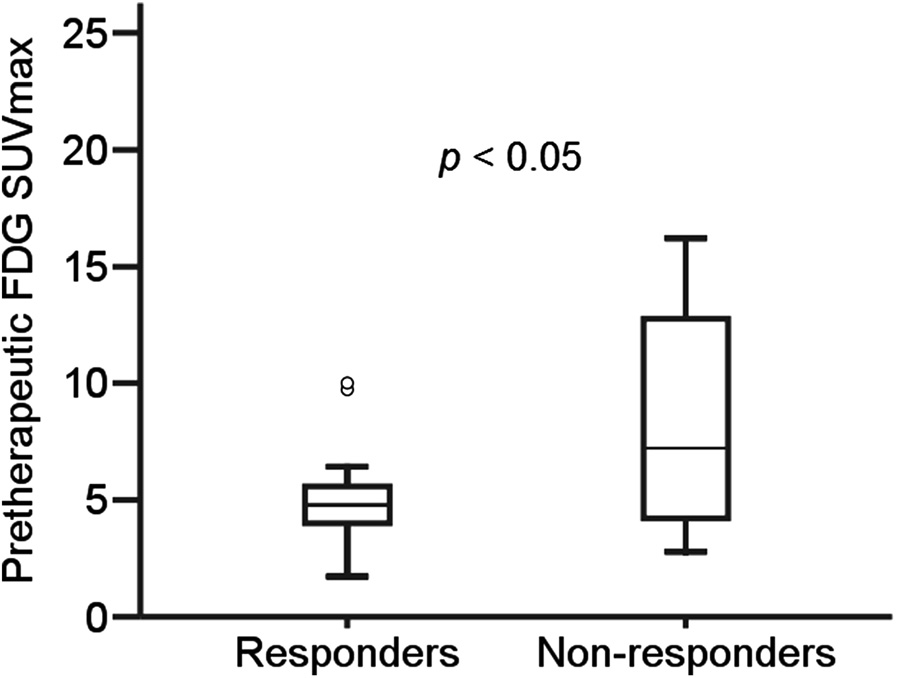
**Comparison of pretherapeutic FDG SUVmax between responders and non-responders.** The median value and the interquartile range are represented by box plot.

The pretherapeutic FDG SUVmax was 4.8 ± 2.0 and 8.5 ± 4.7 (*p* < 0.05) for responders and non-responders, respectively (Figure 3). On PET/CT voxel-based histogram analyses of FDG uptake, FDG demonstrated no significant differences in the skewness, kurtosis, and AUC-CSH between responders and non-responders (Table 2).

**Table 2:** **Comparison of FDG accumulation and intratumoral distribution between responders and non-responders**

	**Responder (** ***n*** **= 26)**	**Non-responder (** ***n*** **= 16)**	***p*** **value**
SUVmax	4.8 ± 2.0	8.5 ± 4.7	<0.05
Skewness	1.07 ± 0.29	1.17 ± 0.40	n. s.
Kurtosis	3.56 ± 0.91	3.99 ± 1.34	n. s.
AUC-CSH	0.30 ± 0.05	0.30 ± 0.06	n. s.

n. s., not significant.

**Figure 4:**
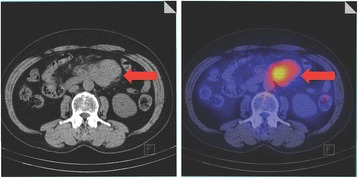
**Representative CT and fused image of the markedly accumulation of** ^**111**^ **In-ibritumomab tiuxetan in the para-aortic lymph node (arrow).** A male patient with B-cell non-Hodgkin’s lymphoma had a standardized uptake value of para-aortic lymph node of 3.81, skewness of 0.813, kurtosis of 2.95, and AUC-CSH of 0.344.

Figure 4 shows a representative image of the markedly accumulation of ^111^In-ibritumomab tiuxetan in the para-aortic lymph node.

On SPECT/CT images, tumor accumulation of ^111^In-ibritumomab tiuxetan was 0.0022 ± 0.0009 and 0.0024 ± 0.0008 %ID/g (not significant) and ^111^In-ibritumomab tiuxetan SUVmax was 2.74 ± 1.43 and 3.29 ± 1.47 (not significant) for responders and non-responders, respectively. In SPECT/CT voxel-based histogram analyses of ^111^In-ibritumomab tiuxetan uptake, the non-responder group showed a more heterogeneous distribution than the responder group. The skewness was 0.58 ± 0.16 and 0.73 ± 0.24 (*p* < 0.05), kurtosis was 2.39 ± 0.32 and 2.78 ± 0.53 (*p* < 0.02), and AUC-CSH was 0.37 ± 0.04 and 0.34 ± 0.05 (*p* < 0.05) for responders and non-responders, respectively (Table 3).

**Table 3:** **Comparison of** ^**11**^ **In-ibritumomab tiuxetan accumulation and intratumoral distribution between responders and non-responders**

	**Responder (** ***n*** **= 26)**	**Non-responder (** ***n*** **= 16)**	***p*** **value**
SUVmax	2.74 ± 1.43	3.29 ± 1.47	n. s.
% ID/g	0.0022 ± 0.0009	0.0024 ± 0.0008	n. s.
Skewness	0.58 ± 0.16	0.73 ± 0.24	<0.05
Kurtosis	2.39 ± 0.32	2.78 ± 0.53	<0.02
AUC-CSH	0.37 ± 0.04	0.34 ± 0.05	<0.05

n. s., not significant.

## Discussion

For ^111^In-ibritumomab tiuxetan, whole-body planar scans are usually performed as two-dimensional planar imaging according to the defined protocol. This may be sufficient to distinguish altered from expected biodistributions. A disadvantage of planar imaging, however, is the lack of three-dimensional information on the tracer distribution [22]. More specifically, the overlap of tumors and normal tissues may obscure tumor boundaries. Three-dimensional images using SPECT/CT may overcome such a problem by fusing functional and morphological images [23,24]. In addition, important roles of the CT component using the SPECT/CT instrument in ^111^In-ibritumomab tiuxetan scans include data acquisition for attenuation correction [25,26], implemented during the quantification of ^111^In-ibritumomab tiuxetan in the lesions.

In this study, a positive correlation was observed between the pretherapeutic FDG SUVmax and accumulation of ^111^In-ibritumomab tiuxetan, which suggested that the higher glucose metabolic activity of lesions, the greater their accumulation of ^111^In-ibritumomab tiuxetan. In this context, glucose metabolism and CD20 antigen expression might parallel each other; thus, potential tumor aggressiveness should be considered when treating ^111^In-ibritumomab tiuxetan accumulating lesions. This may explain why the tumor response is independent of ^111^In-ibritumomab tiuxetan uptake during ^90^Y-ibritumomab tiuxetan therapy. Tumor metabolism of ^111^In-ibritumomab tiuxetan-avid lesions might counter the effect of internal radiation delivered by ^90^Y-ibritumomab tiuxetan, although this is only a speculation at this time.

The time point of 48 h for ^111^In-ibritumomab tiuxetan SPECT/CT can usually be considered appropriate because radiolabeled whole IgGs usually reach peak accumulation 48 h in humans [27,28], although more time points may be needed for in-depth dosimetric studies. Nevertheless, one time point of 48 h is a good compromise to obtain a quantitative value that can be correlated with other variables such as the tumor response.

In radionuclide therapy, non-uniformity of the intratumorally absorbed dose may be a key issue for treatment success or failure [6-9]. We used the three heterogeneity indices of skewness, kurtosis, and AUC-CSH for the analyzing intratumoral distribution of ^111^In-ibritumomab tiuxetan. Higher skewness and kurtosis in the non-responder group, suggesting a more right-skewed distribution and peak of the histogram of voxels, may indicate a heterogeneous intratumoral distribution of ^111^In-ibritumomab tiuxetan on SPECT/CT images. AUC-CSH is thought to correlate with the degree of heterogeneity, i.e., a lower AUC-CSH corresponds with a more heterogeneous distribution [21]. The fact that the non-responder group had a lower AUC-CSH therefore shows that it has a more heterogeneous distribution of ^111^In-ibritumomab tiuxetan than the responder group.

In our study, pretherapeutic FDG SUVmax was significantly correlated with the tumor response on a lesion-by-lesion basis. This result is consistent with a previous report [14], while, in another previous report on ^90^Y-ibritumomab tiuxetan therapy, pretherapeutic FDG SUVmax was not predictive of the tumor response [10]. This may be because they assessed the response on a patient-by-patient basis, and glucose metabolism of each lesion may more favorably predict the response of each lesion. In terms of the long-term outcome, a study reported that a higher pretherapeutic FDG uptake was not correlated with a longer progression-free survival [11]. Pretherapeutic FDG SUVmax in combination with heterogeneity of ^111^In-ibritumomab tiuxetan might enhance the predictive values for tumor response and long-term outcome, which will be clarified in further studies.

A limitation of our study is that SPECT/CT deals with only macroscopic lesions as targets due to its spatial resolution, and so microscopic lesions are outside the scope of consideration. In soft tissue, 90% of the energy emitted by the ^90^Y radionuclide is absorbed within 5 mm. Also, the spatial resolution of SPECT/CT using the LMEGP collimator is only about 9 mm [16]. Hence, the heterogeneity of ^111^In-ibritumomab tiuxetan uptake may be markedly influenced by the tumor size and spatial resolution of SPECT/CT. However, it is clear that SPECT/CT improves the quantification of SPECT alone using techniques such as VOI analysis. In previous ^111^In-ibritumomab tiuxetan studies, the tumor characteristics were mostly evaluated on two-dimensional images or even when three-dimensional images were obtained, and only maximum uptake values reflecting a single voxel with the maximum uptake were considered [29]. The three-dimensional analysis of voxels in this study has an advantage over previous studies and provides new findings.

Another limitation is that we applied a part of the ‘International Workshop Criteria’, a patient-by-patient therapeutic response standard, to evaluate the lesion-by-lesion therapeutic response. This was because no criteria are available for defining response of individual lesions.

## Conclusions

There was a positive correlation between glucose metabolism and ^111^In-ibritumomab tiuxetan accumulation in lesions. Pretherapeutic FDG accumulation was predictive of the tumor response to ^90^Y-ibritumomab tiuxetan therapy. The heterogeneity of the intratumoral distribution rather than the absolute level of ^111^In-ibritumomab tiuxetan was correlated with the tumor response.
